# On Data Selection and Regularization for Underdetermined Vibro-Acoustic Source Identification

**DOI:** 10.3390/s25123767

**Published:** 2025-06-16

**Authors:** Laixu Jiang, Jingqiao Liu, Xin Jiang, Yuezhao Pang

**Affiliations:** Marine Design and Research Institute of China, Shanghai 200011, China; 13501630432@139.com (J.L.); jiangxinxin9925@163.com (X.J.); pangyuezhao518@163.com (Y.P.)

**Keywords:** source reconstruction, near-field acoustical holography, underdetermined data, data selection, regularization

## Abstract

The number of hologram points in near-field acoustical holography (NAH) for a vibro-acoustic system plays a vital role in conditioning the transfer function between the source and measuring points. The requirement for many overdetermined hologram points for extended sources to obtain high accuracy poses a problem for the practical applications of NAH. Furthermore, overdetermination does not generally ensure enhanced accuracy, stability, and convergence, owing to the problem of rank deficiency. To achieve satisfactory reconstruction accuracy with underdetermined hologram data, the best practice for choosing hologram points and regularization methods is determined by comparing cross-linked sets of data-sorting and regularization methods. Three typical data selection and treatment methods are compared: iterative discarding of the most dependent data, monitoring singular value changes during the data reduction process, and zero padding in the patch holography technique. To test the regularization method for inverse conditioning, which is used together with the data selection method, the Tikhonov method, Bayesian regularization, and the data compression method are compared. The inverse equivalent source method is chosen as the holography method, and a numerical test is conducted with a point-excited thin plate. The simulation results show that selecting hologram points using the effective independence method, combined with regularization via compressed sensing, significantly reduces the reconstruction error and enhances the modal assurance criterion value. The experimental results also support the proposed best practice for inverting underdetermined hologram data by integrating the NAH data selection and regularization techniques.

## 1. Introduction

In near-field acoustic holography (NAH) [1,2,3,4,5], the properties and size of the transfer matrix elements between the vibro-acoustic source and the measured pressure are critical for obtaining high-quality results. Many NAH practitioners have stated that the number of measurement points on the hologram should be maximized for high accuracy and resolution, owing to statistical advantages such as using the central limit theorem. However, using many microphones is costly, time-consuming, and challenging to implement in practical systems. Moreover, the overdetermined hologram data do not necessarily improve the precision of the reconstructed data because a large number of measurement points often contain redundancies due to repeated or similar information [6], which increases the condition number of the transfer matrix due to the mutual dependency in the data. Such poor conditions increase the difficulty of inverting the transfer matrix, especially in the presence of measurement noise. Data redundancy implies that the hologram data are not independent, precluding the application of the central limit theorem. Furthermore, most of the hologram field information will be discarded or distorted by the regularization process, degrading the system fidelity. The above considerations imply that during the reconstruction process, the basis of source reconstruction must sometimes be changed from overdetermined hologram data to underdetermined data by using an appropriate rank reduction process.

In addition, sometimes NAH will start with a small number of hologram points that are inadequate compared with the number of target source points. Consequently, the transfer system connecting the vibratory source and the acoustic data will be inherently underdetermined. In such cases, the position of the measurement point is a critical factor affecting reconstruction accuracy. Every hologram point conveys valuable information, although there can be a dependency on hologram data, as in the case of overdetermined data. Therefore, it is vital to maximize each measurement point’s contribution to the reconstruction process in order to overcome the data sparsity inherent in underdetermined hologram data conditions. Moreover, the hologram points containing redundant information must be sorted, as this maximizes the independence of each measurement point, regardless of whether the system is overdetermined or underdetermined. From a mathematical perspective, the column vectors in the transfer matrix established between the source and measurement fields should be as independent of one another as possible. In this regard, it is essential to ensure linear independence between the transfer matrix elements. A low condition number is achieved by selecting appropriate measurement points in the hologram plane.

The inverse problem under consideration must manage the underdetermined transfer matrix, the solution to which is not unique. The classical least-squares method with a pseudo-inverse yields only a minimum norm solution containing a significant error spread in the solution space. Moreover, if the measured data are contaminated with noise—as is always the case—the inverse solution process must be regularized to overcome singularities that cause divergence and the spurious reconstruction problem. One can solve the inverse problem in a least-squares sense using the regularized pseudo-inversion or the Tikhonov regularization (TR) technique with an appropriate regularization parameter. This regularization parameter must be optimized to strike a balance between bias and random errors using appropriate methods, such as generalized cross-validation and the L-curve method. The method best suited to each problem is typically selected on a case-by-case basis [7,8,9,10]. The compressed sensing (CS) algorithm is another regularization approach. It enables the precise reconstruction of a signal from sparsely measured data by solving a convex minimization problem, provided that the signal’s sparsity is predefined. Only a few studies have applied the CS algorithm to NAH using underdetermined sampling to achieve wideband reconstruction [11,12,13,14,15,16]. Most previous studies on the CS algorithm have used the interior-point convex optimization algorithm, implemented in a publicly available commercial toolbox (CVX: MATLAB software (Version 2.1)) for disciplined convex programming), to solve the inverse problem [17]. Hald compared five iterative methods to solve the underdetermined inverse problem. It is concluded that an optimal choice of methodology could yield the iteratively reweighted least-squares (IRLS) algorithm in the traditional Tikhonov frequency range and the iterative zoom-out-thresholding algorithm at higher frequencies [18].

The present study examines the best practice for a reconstruction procedure utilizing underdetermined hologram data, which enhances the resolution of the restored source field using NAH. The test used to choose the best method for selecting hologram points is conducted by applying the effective independence method (EfI), in which data points having the most significant dependency on other points are discarded and the monitoring of singular values (MSV) method, in which the magnitudes of the smallest singular values are monitored by sequential elimination of the measuring points [6,19,20]. Moreover, a comparison is also made with another popular data treatment method used with limited hologram data—the zero-padding (ZP) method, which is employed in the patch holography technique and increases the effective size of the measurement aperture [21,22,23]. In implementing the NAH described in this paper, the inverse equivalent source method [4,5] is employed, utilizing only monopole sources due to its simplicity, computational efficiency, and ability to reconstruct non-separable geometries [13,24]. Furthermore, the three most promising and popular regularization methods are tested by cross-linking them with the data-selection techniques above. The IRLS algorithm [25], which is a CS method known to have good stability [18], TR, and Bayesian regularization [26,27], is employed in the inverse solution process for the underdetermined system established by the optimized measuring points.

To guide readers through the structure of the paper, the organization is as follows. Section 2 introduces the theoretical framework of near-field acoustic holography (NAH), along with two methods for preparing meaningful underdetermined hologram data and the associated regularization techniques. Section 3 presents numerical simulations based on a vibrating plate example, discusses various data selection strategies for constructing underdetermined datasets, and compares reconstruction performance under different regularization approaches. Section 4 validates the proposed method through an experimental setup involving a vibrating plate, demonstrating its practical effectiveness. Finally, Section 5 concludes the study and discusses potential applications and future directions.

## 2. Brief Descriptions in the Theoretical Backgrounds

### 2.1. NAH Based on the ESM

**Figure 1:**
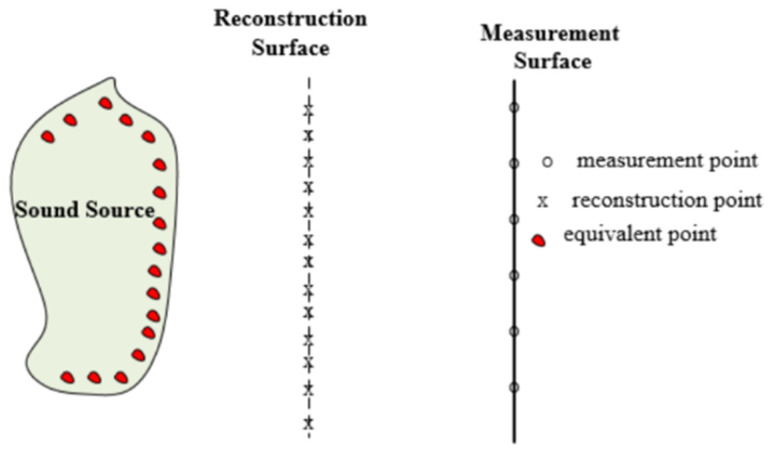
Schematic diagram of equivalent source method.

In an infinite and homogeneous fluid medium, the acoustic pressure at any field point can be expressed by the superposition of sound fields generated by a series of equivalent point sources distributed inside the structure. One can use all different pole representations of a source for the equivalent sources, but only the monopole sources are employed in this work for simplicity. The principal diagram is shown in Figure 1.

In the ESM, the measured pressure vector $P^{H}$ and the source field pressure vector $P^{R}$ can be represented, respectively, in matrix form as [28]

(1) $$P^{H}=j\rho \omega G_{P}^{\Gamma H}Q^{\Gamma },$$

(2) $$P^{R}=j\rho \omega G_{P}^{\Gamma R}Q^{\Gamma },$$

where ω is the angular frequency, ρ is the density of the medium, $Q^{\Gamma }$ is the strength vector of the equivalent sources located at the equivalent source surface Γ, $G_{P}^{\Gamma H}$ is the free-field Green’s function ($=e^{-jkR_{n}^{\Gamma H}}/4\pi R_{n}^{\Gamma H}$) between the measured pressure and the equivalent source surface Γ, and $G_{P}^{\Gamma R}$ means the Green’s function between the source field pressure and the equivalent source surface Γ. $R_{n}^{\Gamma H}$ and $R_{n}^{\Gamma R}$ represent the distance from the measured and source field points to the equivalent source points, respectively. When Equation (2) is substituted into Equation (1), the field pressure can be related directly to the source field pressure as

(3) $$P^{H}=G_{P}^{\Gamma H}{\left[{\left(G_{P}^{\Gamma R}\right)}^{H}G_{P}^{\Gamma R}\right]}^{-1}{\left(G_{P}^{\Gamma R}\right)}^{H}P^{R}\equiv WP^{R},$$

where W is the transfer matrix correlating the measured field and source field, and the superscript “*H*” signifies the Hermitian operator. Then, using the singular value decomposition, the source field can be reconstructed as

(4) $$P^{R}=W^{+}P^{H},$$

where $W^{+}(=V\Lambda^{-1}U^{H})$ denotes the pseudo-inverse of W, Λ is the diagonal matrix of singular values, and U, V represent the unitary orthonormal vectors, called the left and right singular vectors, respectively. Due to the singularity, the process of Equation (4) often diverges, so we need to introduce the regularization. This work denotes $P^{R}$ as the sound pressure value on the reconstruction plane, which is very close to and above the actual vibrating panel surface. One can recall that the vibrating surface is often too irregular, making it difficult to measure the surface vibration accurately. Sometimes, the surface is covered with a porous or furred material, so the definition of the surface location is obscure. In many cases, a hypothetical surface surrounding the actual surface is helpful in the forward prediction of the sound field because of its simple shape and smaller area if it is very near to the actual surface.

### 2.2. Preparation of Meaningful Underdetermined Hologram Data

As discussed above, there can be excessive or similar information in the overdetermined hologram data, which increases the correlation between column vectors of the transfer matrix. This means that the overdetermination does not always guarantee the linear independence of the transfer matrix. Consequently, the matrix will have a large condition number, making the inversion of the transfer matrix a challenging task. For the underdetermined hologram data, the measured pressure at each hologram point should be meaningful and critical for source reconstruction, given the sparsity of the available data. In this context, hologram points that provide repeated information should be discarded, even though the data is further reduced. Even for the overdetermined case, the same is true. From a mathematical point of view, it means that each column vector in the established transfer matrix between source vibration and hologram pressure should be independent as far as possible. Therefore, the best selection of hologram points for a given target number of hologram data is necessary to assure the linear independence of the transfer matrix and low condition number level. If one starts the selection process from the much-overdetermined mother population, the sequential data reduction can be performed by monitoring the singularity index. If the selection process begins with a fixed, small, and evenly spaced set of data points, one needs to expand the transfer matrix using known data or by setting it to zero [21,22,23].

#### 2.2.1. Sequential Elimination of the Most Dependent Positions

The effective independence method [6,19,20] helps rank the sensor locations according to their contribution to the linear independence of the transfer matrix in NAH. The contribution of each measurement location to the linear independence of the field pressure for a given measurement setup is represented by the EfI value, defined as follows:

(5) $$E=\mathrm{diag}(U_{C}U_{C}^{H}).$$

Here, the operator ‘diag’ means the vector consists of diagonal elements of the involved matrix and $U_{C}$ denotes the matrix spanned by the first C column vectors of the left singular vector U. The range of the EfI value is 0–1, and one can use the frequency average EfI value for a range of frequencies [6,19]. The higher the EfI value, the higher the linear independence. The best hologram points can be selected from the initial candidate points, usually in a uniformly spaced layout, by sequentially deleting the measurement points with the smallest EfI values. One should note that the selected positions are not globally optimal, but optimal for a given number of sensor positions. By completing the iterative elimination of the most dependent column vectors, one ends up with the target number of overdetermined points or even achieving an underdetermined number of hologram points. Hereafter, this method is referred to as the EfI technique for simplicity.

#### 2.2.2. Sequential Elimination of Measuring Points Yielding the Smallest Singular Values

This method combines the chosen field points to result in a small condition number [6]. Because the singular values can be thought of as an index of the singularity of the transfer matrix, one can delete the transfer matrix element related to the small singular value as a rank-deficient one. First, one should generate a mother population of m candidate field points and calculate all possible transfer matrices relating *n* source points to (m − 1) field points. This process can be performed efficiently by manipulating $G_{P}^{\Gamma R}$ and $G_{P}^{\Gamma H}$ in Equation (3). Then, take the average of condition number over the frequency range of interest and select any combination with the minor average. Finally, repeat the procedure M times to select (m − M) field points until the number meets the predetermined or allowed number of points. For simplicity, this method is commonly referred to as the MSV method.

#### 2.2.3. Expansion of the Patch Hologram Data with Zero Padding

When the number of hologram points is far smaller than the source points, one can expand the hologram data by allocating zeroes at the extended area outside and inside the original hologram plane. Such an iterative method using the patch extension with zero padding is based on the measured pressure $P^{H}$ on $\Gamma_{M}$ to extrapolate the pressure on $\Gamma_{E}$. First, if the outside of the patch zone is to be extended, the hologram data $P_{1}^{H}$, comprising the measured pressure $P^{H}$ in $\Gamma_{M}$ and zeros in $\Gamma_{E}$, are used to reconstruct the whole field $P_{2}^{H}$ on $\Gamma_{M}\cup \Gamma_{E}$. Then, the patch pressure $P_{2}^{H}$ is replaced by the measured initial pressure $P^{H}$ on $\Gamma_{M}$ as the input for the next iteration. The process is then repeated *n* times until the desired convergence ${\left\|(P_{n}^{H}-P_{n-1}^{H})/P_{n-1}^{H}\right\|}_{2}\leq \varepsilon$ is reached for a predetermined convergence criterion ε. Many previous experimental and simulation works have demonstrated that the results of such data expansion tend to be stable after a certain number of iterations [29]. In general, the iteration is terminated once this predetermined convergence criterion ε falls below the prescribed tolerance of ε = 1 × 10^−4^, or when the number of iterations reaches seven, whichever occurs first. Additionally, it has been demonstrated that the Tikhonov regularization technique can be effectively applied during iterative reconstruction [30].

Hereafter, this method is referred to as the ZP method for simplicity.

### 2.3. Regularization of the Inverse Operation Using the Underdetermined Hologram Data

The transfer matrix contains the non-propagating wave components, and noise contamination cannot be avoided in practice. To prevent the divergence phenomenon in the matrix inversion, one should somehow use proper filtering or regularization to overcome the ill-posedness of the transfer matrix. In the present work, regularization techniques are tested by combining them with hologram data selection methods in a cross-linked manner to enhance reconstruction accuracy.

#### 2.3.1. Tikhonov Regularization Adapting the GCV

The estimation $Q^{\Gamma }$ is often obtained via the Tikhonov-regularized Moore–Penrose pseudo-inverse. The objective function includes two aspects. One is the residual norm, and the other is the discrete norm with the regularization parameter expressed as follows:

(6) $$\mathrm{Minimize}\left\{{\left\|j\rho \omega G_{P}^{\Gamma H}Q^{\Gamma }-P^{H}\right\|}_{2}^{2}+\lambda^{2}{\left\|LQ^{\Gamma }\right\|}_{2}^{2}\right\}.$$

Here, λ denotes the regularization parameter and L is an arbitrary matrix. When L is the identity matrix, the corresponding minimum value can be written as

(7) $$Q^{\Gamma }={\left({\left(G_{P}^{\Gamma H}\right)}^{H}G_{P}^{\Gamma H}+\lambda^{2}L^{H}L\right)}^{-1}{\left(G_{P}^{\Gamma H}\right)}^{H}P^{H}.$$

Using the SVD technique, the regularized solution $Q^{\Gamma }$ is given by

(8) $$Q^{\Gamma }=\sum_{i=1}^{n}\frac{\Lambda_{i}^{2}}{\Lambda_{i}^{2}+\lambda^{2}}\frac{u_{i}^{H}P^{H}}{\sigma_{i}}v_{i},$$

where $G_{P}^{\Gamma R}=U\Lambda V^{H}$.

The critical issue of Tikhonov regularization is to find an optimal wave-vector filter shape by an optimal Tikhonov parameter or iteration number. There are various searching tools for an optimal regularization parameter, such as the Morozov discrepancy principle [30], the generalized cross-validation technique (GCV) [31], the L-curve method [9], etc., [32]. Although the best choice of the parameter extraction method depends on the problem, it is known that the difference in residual error level is not very significant [33]. In this work, the GCV method is used to select the Tikhonov regularization parameter (*λ*) in a data-driven manner. The GCV algorithm automatically identifies the value of *λ* that best balances reconstruction accuracy and stability for our NAH problem. This practical approach avoids subjective trial-and-error tuning by using the measured hologram data to determine an optimal trade-off. In essence, it would over-amplify measurement noise if *λ* is too small, whereas a too significant value would over-smooth important details of the acoustic field. By minimizing the GCV criterion, we effectively filter out noise while preserving true acoustic features, ensuring a stable inversion without sacrificing spatial resolution.

#### 2.3.2. Statistical Regularization Based on the Bayesian Technique

The Bayesian regularization criterion has been proposed as a selection method of the regularization parameter for the sound field reconstruction [26]. The idea originates from the inductive reasoning of probability theory, which combines sample information with non-sample information. It is particularly suitable for solving general inverse problems by deducing a solution from small samples and applying it to the whole or particular samples by inference. The unknown acoustic quantity of interest is taken as the random variable to be combined with the physical and probabilistic information to construct the optimization function. Then, by solving the conditional probability, the optimal solution of the unknown quantity can be obtained [26]. In this way, one can obtain the optimal regularization parameter by minimizing the following function:

(9) $$J(\lambda^{2})=\sum_{k=1}^{n}\ln\left(\Lambda_{k}^{2}+\lambda^{2}\right)+\left(n+2\right)\ln\left(\frac{1}{n}\left(\sum_{k=1}^{n}\frac{{\left|y_{k}\right|}^{2}}{\Lambda_{k}^{2}+\lambda^{2}}\right)\right).$$

Here, *n* is the number of the hologram data, and $y_{k}$ denotes the kth element of a vector as given by

(10) $$y_{k}=U^{H}P^{H}.$$

It is worth noting that in the Bayesian regularization approach, the key parameter is the expected noise level in the measurements (i.e., the noise floor). We estimated this noise floor by analyzing low-signal (background) measurements from the hologram data, and we used that estimate to set the algorithm’s assumed noise variance. Practically, this means the algorithm treats any residual pressure below that threshold as noise and does not attempt to fit it. By choosing the threshold based on actual sensor noise, we ensure that the Bayesian inversion focuses on reconstructing the genuine acoustic field and does not chase spurious noise-induced fluctuations. This noise-informed regularization strategy makes the source reconstruction robust against measurement uncertainty in our NAH experiments.

Hereafter, this statistical method is referred to as the BR method for simplicity.

#### 2.3.3. Regularization Using the Data Compression Technique

As mentioned above, most methods for solving the inverse problem of underdetermined systems use the interior-point convex optimization algorithm [11,12,13,14,15,16]. All these methods are sensitive to the selected constraint parameter, representing the noise floor [18]. Also, there is no fast and automated solution method for parameter selection. The iteratively reweighted least-squares (IRLS) method is a type of sparse reconstruction method based on the data compression technique, a simple and potentially fast iterative algorithm for solving the inverse problem using the L1 norm [18,25]. When the equivalent point source strengths are sparse or close to the sparse condition, the unknown source strength column vector $Q^{\Gamma }$ in Equation (1) can be directly solved by a sparsity promoting one-norm regularization as

(11) $$\underset{Q^{\Gamma }}{Minimize}\left\{{\left\|P^{H}-j\rho \omega G_{P}^{\Gamma H}Q^{\Gamma }\right\|}_{2}^{2}+\theta {\left\|Q^{\Gamma }\right\|}_{1}\right\},$$

where θ denotes the regularization parameter. The basic idea of the IRLS algorithm is introducing a weighting matrix *W* to the norm solution and converting the constraint term ${\left\|Q^{\Gamma }\right\|}_{1}$ into ${\sum_{i}^{n}W_{i}}^{-2}{\left|q_{i}^{\Gamma }\right|}^{2}$. Then, Equation (11) can be rewritten as [18]

(12) $$\underset{Q^{T}}{Minimize}\left\{{\left\|P^{H}-j\rho \omega G_{P}^{\Gamma H}Q^{\Gamma }\right\|}_{2}^{2}+\theta \sum_{i}^{n}\;W_{i}^{-2}{\left|q_{i}^{\Gamma }\right|}^{2}\right\},$$

where $W_{i}^{\left(l\right)}=\sqrt{\left|q_{i}^{\Gamma }\right|}$. Therefore, the $W_{i}^{\left(l\right)}$ value is calculated based on the results from the last approximation $q_{i}^{\Gamma (l-1)}$. Arranging the weights $W_{i}^{\left(l\right)}$ on the elements of a diagonal matrix $W^{\left(l\right)}$ allows Equation (12) to be expressed as follows:

(13) $$\underset{Q^{\Gamma }}{Minimize}\left\{{\left\|P^{H}-j\rho \omega G_{P}^{\Gamma H}Q^{\Gamma }\right\|}_{2}^{2}+\theta {\left\|{\left(W^{\left(l\right)}\right)}^{-1}Q^{\Gamma }\right\|}_{2}^{2}\right\}.$$

One can easily recognize Equation (11) as equivalent to the standard-form of Tikhonov regularization, for which the solution of Equation (13) can be obtained

(14) $$Q^{\Gamma (l)}=W^{\left(l\right)}{\left({\left(F_{P}^{H(l)}\right)}^{H}F_{P}^{H(l)}+\lambda^{2}L^{H}L\right)}^{-1}{\left(F_{P}^{H(l)}\right)}^{H}P^{H},$$

where $F_{P}^{H(l)}=G_{P}^{\Gamma H}W^{\left(l\right)}$. Equation (12) can be further calculated by the singular value decomposition of $F_{P}^{H(l)}$. The optimal λ value can be selected using iteration parameter selection methods such as the GCV, L-curve method, etc. As the first step of the iteration, $W^{1}$ is set as the unit diagonal matrix. The iteration is stopped when the change in the results after the last two iterations is tiny, namely,

(15) $${\left\|Q^{\Gamma (l)}\right\|}_{1}/{\left\|Q^{\Gamma (l-1)}\right\|}_{1}>\delta ,$$

where δ<1. The number of required iterations depends on the degree of underdetermination of the problem and the frequency. According to the previous simulation test, it is known that the result will converge after a small number of iterations [18]. In the model of this work, δ is set to δ = 1 × 10^−4^.

It is worth noting that in the IRLS-based (iteratively reweighted least squares) data compression method, we introduced a stopping tolerance as the key regularization control. This tolerance was selected by observing when further IRLS iterations no longer yielded significant improvements in the reconstruction. Specifically, once the difference between the measured hologram and the IRLS-reconstructed hologram dropped to the order of the measurement noise floor, we terminated the iterations. Halting at this noise-informed threshold prevents the algorithm from overfitting to noise: it effectively compresses the data representation by ignoring negligible residual components and retaining only the meaningful acoustic information. This practical stopping criterion ensures that the reconstructed sound field remains compact and physically meaningful, reflecting the actual source characteristics without amplifying noise.

Hereafter, this data compression method is referred to as the IRLS method for simplicity.

## 3. Numerical Test

### 3.1. Test Model and Selection of Measurement Points

**Figure 2:**
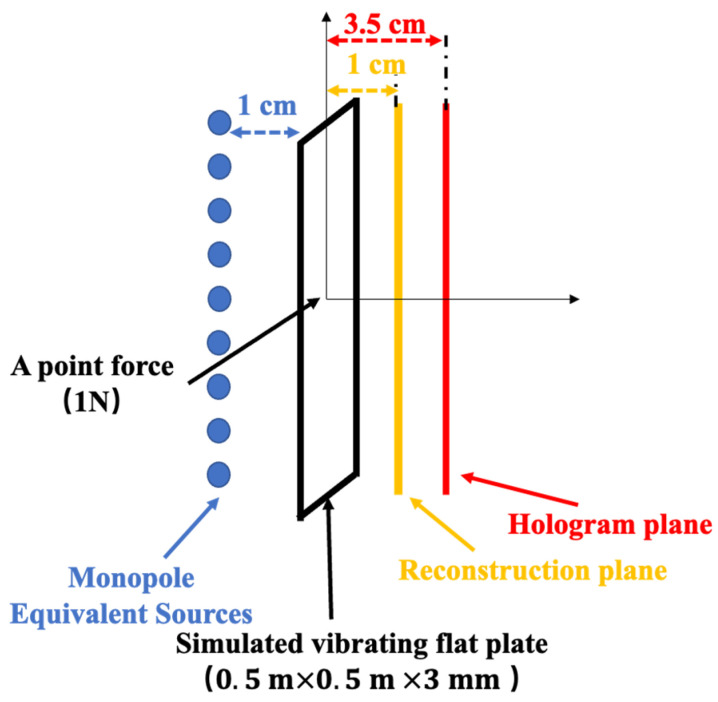
Schematic diagram of the numerical simulation setup.

A vibrating rectangular steel plate of 0.5 m × 0.5 m × 3 mm in a rigid flat baffle was taken as the test example of the source to reconstruct. It should be noted that due to the rigid baffle configuration used in this study, the hologram field does not exhibit the typical edge decay observed in free-field measurements. This specific setup represents bounded source conditions commonly encountered in practical applications and allows for a focused comparison of data selection and regularization methods. A point force drives the plate with an amplitude of 1 N acting at the center. The hologram, reconstruction, and equivalent source plane possess the same dimension as a flat plane. The three planes are located at 3.5 cm, 1 cm above the plate, and 1 cm below the plate, respectively, as shown in Figure 2.

In this test example, 64 points on the reconstruction plane were used as the target source positions. Prior to introducing the data-selection procedure, the fundamental vibrational characteristics of the clamped plate were identified via the Rayleigh method. The first seven natural frequencies were 59 Hz (1, 1), 147 Hz (2, 1)/(1, 2), 235 Hz (2, 2), 385 Hz (2, 3)/(3, 2), and 528 Hz (3, 3). These values are summarized in Table 1 and will serve as a reference in Section 3.3. The spatial pressure pattern of the (2, 3) mode is depicted in Figure 4; full contour plots of the other modes can be obtained from the authors upon request.

**Figure 3:**
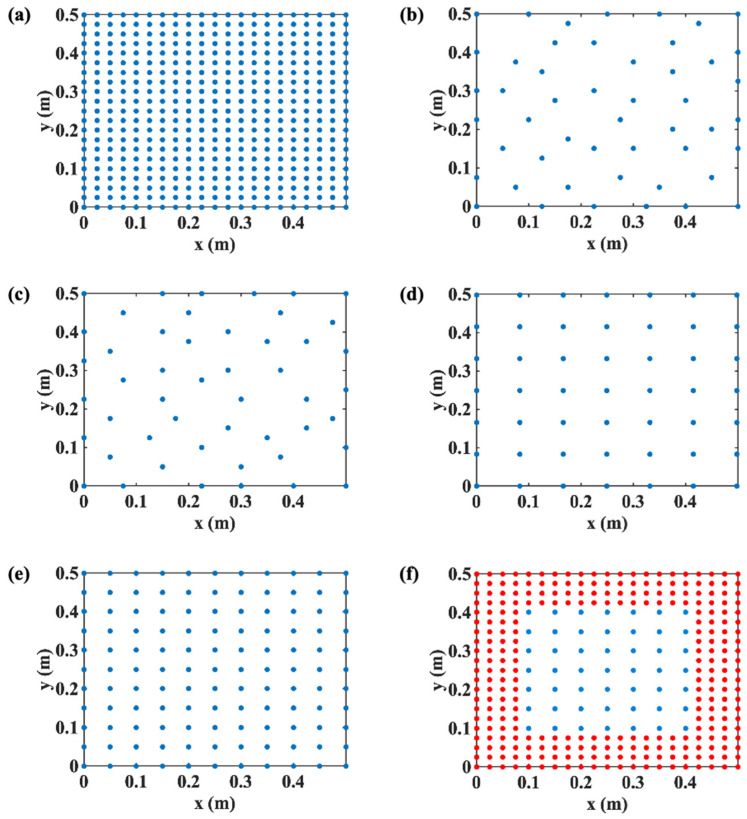
Distribution of measurement points at the hologram plane: (**a**) initial 441 evenly distributed candidate points; (**b**) 49 measurement points selected by the EfI method; (**c**) 49 measurement points selected by the MSV method; (**d**) evenly spaced 49 points; (**e**) evenly spaced 121 points; (**f**) evenly spaced 49 points with 272 points of zero-padding. 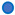 is the measurement points, and 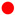 is the padding points.

As the initial candidate hologram data points, 441 measuring points were uniformly spaced by 2.5 cm, as depicted in Figure 3a. As can be seen in Figure 3b,c, the measurement points were reduced to 49 points by applying the frequency-averaged EfI technique and the MSV method, respectively, within the frequency range of 60–600 Hz, where 28 uniformly distributed frequencies were selected for averaging. Additionally, two measurement point sets consisting of 49 and 121 points, as shown in Figure 3d,e, were used to study the validity of the selecting methods of the measurement points. The purpose of preparing the measurement set in Figure 3e is to investigate how many measuring points can be eliminated by the EfI and MSV methods while maintaining the same singularity factor with the original evenly spaced measurement set. In Figure 3f, one can see that the underdetermined hologram data confined in a small zone was expanded with zero padding. The initially given, i.e., assumed as measured, 49 hologram data were evenly distributed with 5 cm grid spacing inside the 0.3 m × 0.3 m area. The outside of this hologram area was filled with additional points assigned as zeroes with a spacing of 2.5 cm. As an indicator of the degree of the singularity of the transfer matrix **W** and the numerical stability of the inversion process, the singularity factor, $S_{F}$, and condition number, CN, are given by

(16) $$S_{F}=tr\left[diag(\Lambda {)}^{-2}\right]$$

(17) $$CN=\Lambda_{max}/\Lambda_{min}.$$

**Figure 4:**
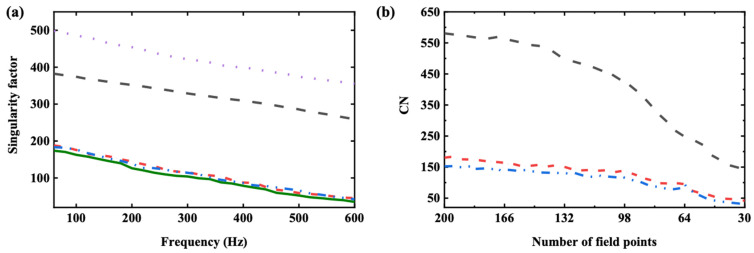
(**a**) Calculated singularity factor of different measurement sets in Figure 3b–f, (**b**) calculated condition number of different measurement sets in Figure 3b–d by progressively reducing the number of field points. The meaning of each curve is as follows: 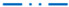, selected by the EfI method; 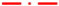, selected by the MSV method; 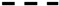, evenly spaced points; 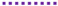, evenly spaced 49 points with zero padding; 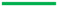, evenly spaced 121 points.

The singularity factor $S_{F}$ was calculated for five measurement sets, and the results were compared, as illustrated in Figure 4a. Also, the condition number of the transfer matrices in the hologram measurement points, which were selected using three data selection methods at 385 Hz, is shown in Figure 4b.

In Figure 4a, one can find that the singularity factors generated by the hologram measurement points selected by the EfI and MSV methods were similar or much smaller than the evenly spaced hologram and the points obtained by the ZP method. This resulted in a small reconstruction error because the same number of points, i.e., 49, were used on the hologram plane in the given frequency range. Compared with the hologram having evenly spaced 121 points, which is a typical overdetermined case, the EfI and the MSV methods yield a very similar magnitude of the singularity factor. This reveals that the underdetermined hologram data optimized by the EfI or MSV method can overcome the disadvantages of under-sampling. That is, the resulting singularity factor is similar to the overdetermined system. In Figure 4b, one can find that the condition number generated by the hologram measurement points selected by the EfI and MSV methods was, overall, much smaller than the ZP method with the number of the same points. Also, the condition number of the transfer matrix decreases with the reduction in field points because the redundant information is removed from the matrix, so the condition number naturally decreases with the decrease in matrix size. Another phenomenon that can be observed is that there is a small peak in CN when the EfI method is employed, which is consistent with Ref [6]. This condition occurs when the number of effective field points exceeds 64, gradually decreasing from 200 to 30. The number of points is the demarcation between overdetermined and underdetermined conditions. The condition number starts to increase because the zero bound increases due to the inherent character of the matrix system.

### 3.2. Comparison of the Layout of the Measurement Points

As discussed above, the hologram data selected by the EfI or MSV method can yield a far smaller singularity factor and condition number than the evenly spaced points. One can confirm the convergence of the inverse operation with such underdetermined hologram data. In the viewpoint of the accuracy of the restored source, one is concerned about the fidelity measures like the average error norm and the similarity of the restored source strength distribution to the original one. Such fidelity performance measures are investigated for different hologram point layouts. The parameter settings of the simulated object and the hologram, reconstruction, and equivalent source surface are the same as those in the preceding section. For restoring the 64 points at the reconstruction plane, three configurations with 441 points each are considered the mother populations or initial candidate points in the hologram plane. As in the former test, 49 points are the target number of points in the hologram plane, far smaller than the number of reconstruction points. In two populations, the field points are removed one by one, or group by group, by monitoring the changes in condition number and EfI value until the target number of points is reached. In the third population, the 49 underdetermined hologram data are evenly distributed over the plane. In the test, the (2, 3) mode of the plate vibration at 385 Hz is considered an example. Random noise with a signal-to-noise ratio (SNR) of 20 dB is added to the pressure data to simulate the actual measurement with noise contamination. All the reconstruction processes are calculated without regularization. Two measures quantify the reconstruction error: the root-mean-squared error norm, $e_{rms}$, expressed in percentage, and the modal assurance criterion, MAC. They are defined as

(18) $$e_{rms}=\frac{{\left\|P^{R}-{\hat{P}}^{R}\right\|}_{2}}{{\left\|P^{R}\right\|}_{2}},$$

(19) $$MAC=\frac{{\left|P^{R}{\hat{P}}^{R}\right|}^{2}}{{\left|P^{R}\right|}^{2}{\left|{\hat{P}}^{R}\right|}^{2}},$$

where $P^{R}$ and ${\hat{P}}^{R}$ denote the true and the reconstructed pressures, respectively. The MAC implies the shape correlation between the measured and reconstructed sound pressure levels.

**Figure 5:**
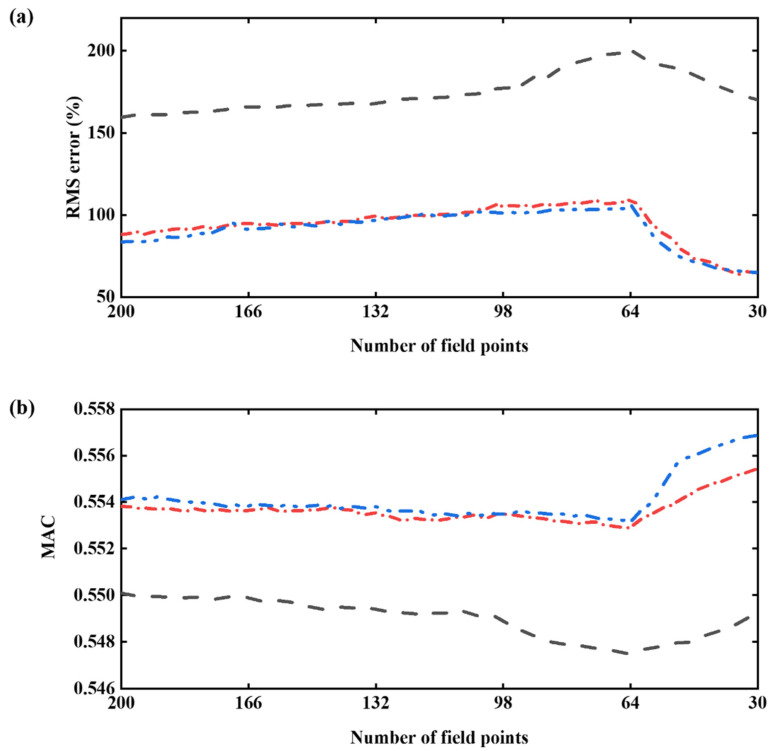
Change in reconstruction condition varying the number of hologram data: (**a**) RMS error norm, (**b**) MAC value. The meaning of each curve is as follows: 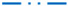, selected by the EfI method; 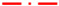, selected by the MSV method; 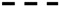, evenly spaced 49 points.

The average error norm $e_{rms}$ and MAC value varying the number of hologram data are shown in Figure 5. The number of hologram data varies from 200 to 30 points, passing through the source number 64 and the preset target number 49.

As mentioned in Figure 4b, the condition number of the transfer matrix decreases as the field points are eliminated by the repeated application of the reduction methods. One can recall that the singularity factor of the transfer matrix determined by the EfI technique and MSV method is much smaller than that of the evenly distributed points. It leads directly to the results, as shown in Figure 5a,b. The underdetermined hologram data optimized by the EfI or MSV method yields better reconstruction accuracy and larger MAC value than the evenly distributed, underdetermined system. From the mathematical point of view, the strong correlation between vectors in a matrix leads to a worsened ill-condition of the matrix. Consequently, one must discard a part of the vectors that leads to the strong correlation among the vectors constituting the transfer matrix, thus obtaining a stable one with a good condition number.

In Figure 5, one can also find a small peak in $e_{\mathrm{rms}}$, and a small through in MAC value. This is due to the sudden increase in CN at the same number of field points as the source points, which is 64. Then, shortly after passing the point, the reconstruction error gradually decreases as field points drop to the underdetermined stage using the EfI or MSV methods. This is caused by the fact that the problem has fewer constraints than the overdetermined situation, reducing the difference between the maximum and minimum singular values. This means that the reconstruction result using the underdetermined data improves on that using the overdetermined data. One should note that this phenomenon does not happen in the usual reconstruction from the evenly spaced hologram points. It is worth remembering that the data reduction initially started from a large set of overdetermined candidate field points, so the underdetermined condition, which produces a significant enhancement of the reconstruction, is selectively chosen from this large overdetermined population [6]. The critical point is that the reconstruction results of underdetermined data can achieve the same or even better reconstruction resulting from relatively sufficient hologram data, as supported by the findings in the preceding section. One can notice that the $e_{\mathrm{rms}}$ has a continuous decreasing trend, while the MAC value increases monotonically when the number of points decreases beyond the number of reconstruction points. One may suggest a further reduction in hologram data. However, in such a case, the improvement of reconstruction errors or MAC value does not seem prominent compared to the beginning of the underdetermined stage. This might be because the current redundant measuring data contains too little information, and the distance between the measuring points is close to the Nyquist criterion limit. The improvement in condition number and the impairment of fidelity are offset by each other.

### 3.3. Reconstruction of Source Field Using the Regularization

**Figure 6:**
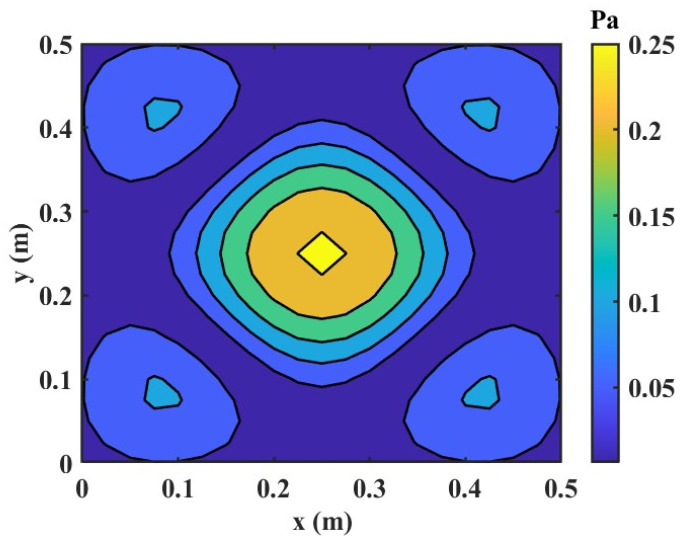
Numerically obtained pressure distribution on the reconstruction plane (f = 385 Hz).

Although there are many regularization techniques, the most popular and promising techniques for the NAH are chosen for comparison purposes: TR, BR, and compressed sensing techniques. Then, the performances are combined with the preceding hologram point selection methods in a cross-linking way. The parameter settings and three data sets are the same as in Section 3.1. A total of 49 hologram data are used as the target number of points to reconstruct 64 points at the reconstruction plane. Random noise with a signal-to-noise ratio (SNR) of 20 dB is added to the pressure data to simulate a practical measurement contaminated with noise. The considered plate is excited at 385 Hz because it coincides with an important plate resonance, which corresponds to the resonance frequency of (2, 3) mode. Figure 6 shows the numerically obtained pressure distribution on the reconstruction plane at 385 Hz.

**Figure 7:**
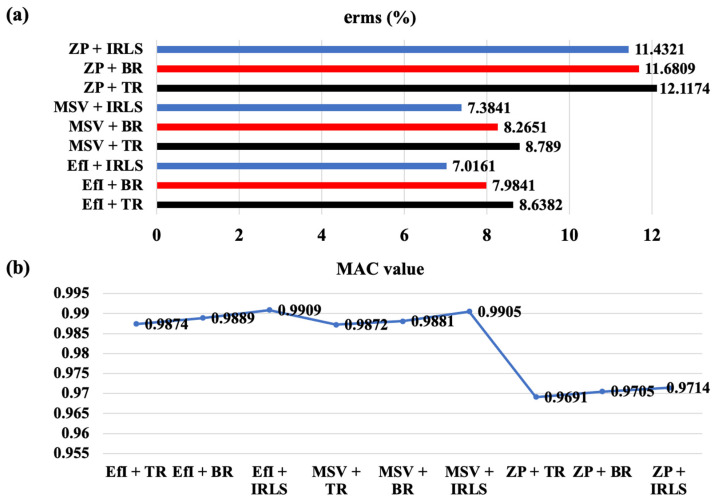
Summary of the reconstruction error in terms of (**a**) error norm, (**b**) MAC value (f = 385 Hz).

Figure 7 summarizes reconstruction results from the cross-linked combination of the three hologram point selection methods and the three regularization methods. One can find that the underdetermined hologram data optimized by the EfI technique or MSV method produces better reconstruction results than the patch with zero padding points, regardless of the regularization algorithm. This is because the transfer matrices after applying the EfI technique or MSV method are better conditioned and more independent than that by the patch with zero padding points. The EfI technique yields a slightly better result than the MSV method, but the difference is acceptably small. It is not difficult to understand that the reconstruction result of the patch with the zero-padding technique is not very good because one cannot guarantee a high degree of independence among the original hologram data. In addition, the probability of overlapping in regenerated data increases after iterative expansion, so the independence between data decreases. Then, it results in the increased condition number of the transfer matrix, as shown in Figure 4a. This situation becomes severe in the presence of noise, in particular. Therefore, investigating the usefulness of the regenerated data is worth considering. Even if regularization is employed for the inverse filtering of the regenerated data, significant work remains necessary to be performed to ensure high-quality reconstruction results. By comparing the three regularization algorithms from the viewpoint of MAC value, the IRLS and BR methods yield much better results than the TR for all different layouts of hologram data. Overall, the IRLS works best, especially when it is combined with the EfI technique. The mutual independence between the column vectors of the transfer matrix is reflected in the compressed sensing technique to promote the sparsity of the compressed matrix, promoting more sparse reconstruction. One can conclude that the EfI or the MSV method can be an excellent reconstruction strategy when used in conjunction with the CS algorithm. With a little bit more error, one can consider the BR as acceptable, too.

**Figure 8:**
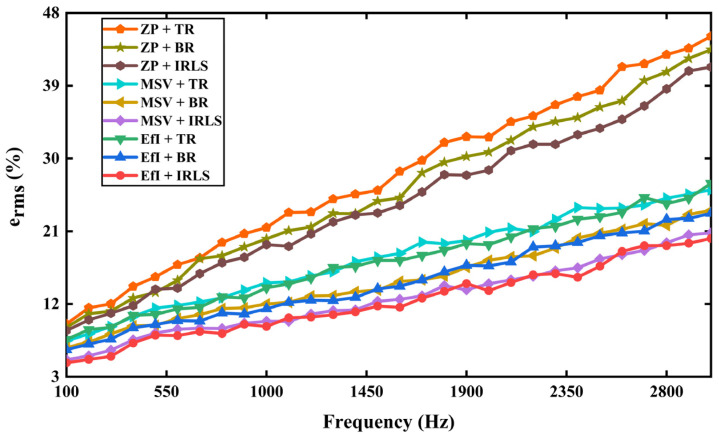
A comparison of the reconstruction error in using the three regularization algorithms cross-linked with three sets of different hologram distributions.

The reconstruction performance in the frequency band of 0.1–3 kHz is tested, as shown in Figure 8. One can see that the reconstruction error of the hologram data selected by the patch with zero-padding technique bears a more significant error than the other data selection techniques, regardless of the regularization methods. Also, it is noted that the combination of the EfI or MSV technique with the IRLS method of compressed sensing technique yields a minor error with underdetermined hologram data for all frequencies. It can be explained again that the EfI and MSV methods can enhance the independence between the column vectors of the transfer matrix, resulting in promoted sparsity of the transfer matrix. The results show that the optimized underdetermined hologram data using EfI and MSV methods are of excellent quality and can carry out the corresponding inverse problem solution.

**Figure A1:**
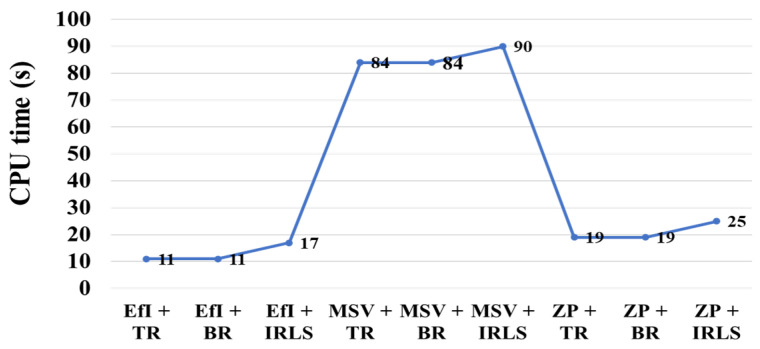
CPU time in generating the results in Figure 8.

On the other hand, one can observe that the difference between the results of the three algorithms performed at low frequencies is not as significant as it is at high frequencies. As the frequency increases, one can recall that more measuring points need to be added to satisfy the sampling theorem as much as possible in NAH. The IRLS method yields the difference between the BR and TR methods in terms of frequency and performs sparse reconstruction based on the same number of measurement points. A comparison of the calculation time using MATLAB for Figure 8 is summarized in Figure A1 in the Appendix A. The data reveal that the EfI technique combined with any regularization method is the fastest in general.

### 3.4. Effects of SNR and Measuring Distance

The influence of SNR and measuring distance of the hologram plane on the reconstruction performance are also investigated. The analysis frequency is 385 Hz, and the test parameters are consistent with those in Section 3.1, except for the SNR and measuring distance. First, the SNR is changed from 10 to 50 dB in 5 dB steps while maintaining a measuring distance of 0.035 m. Second, the hologram distance from the source surface is adjusted from 0.03 to 0.15 m with a 0.01 m step, while setting the SNR as 20 dB. In the SNR test, one can recall that the SNR range setting of 10 to 50 dB is sufficient to see the effect on the inversion process, and the maximum measuring distance of 0.15 m is also satisfactory for NAH, which is close to the one-sixth of the wavelength of the analysis frequency.

**Figure 9:**
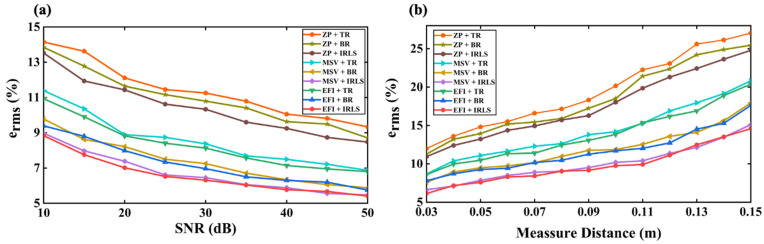
A comparison of reconstruction error in using the three regularization algorithms cross-linked with three sets of different hologram distributions: (**a**) varying the SNR (f = 385 Hz, d = 0.035), (**b**) varying the measuring distance (f = 385 Hz, SNR = 20 dB).

Figure 9a shows the reconstruction errors of the three algorithms when three sets of hologram distributions are chosen with SNR variation at 385 Hz. One can observe that the reconstruction error decreases with the increase in SNR, as expected in general. Additionally, regardless of the algorithm used to process the underdetermined system, the reconstruction result of hologram data optimized by EfI or MSV methods is consistently superior to that of the patch with zero padding points. The CS algorithm can provide the best accurate reconstruction to process the optimally selected underdetermined hologram data than the BR with SNR variation. In any case, TR-GCV regularization performs the worst. Figure 9b shows the reconstruction error versus measuring distance. Similar results are observed for the SNR test. In this work, the IRLS algorithm is used to solve the inverse problem of the underdetermined system optimized by the EfI technique. Still, other compressed sensing algorithms would also achieve comparable results. The core problem is that the EfI or the MSV method can improve sparsity to a certain extent and promote the work of data compression.

## 4. Experimental Test

### 4.1. Test Setup and Method

A steel plate with a dimension of 0.7 m × 0.7 m × 3 mm was used as the sound source, which is driven at the center by a shaker, and its boundary was fixed. The excitation signal was synthesized from a series of sinusoidal signals for a 200–2000 Hz range with a frequency interval of 100 Hz. The hologram and reconstruction planes were located 10 cm and 5 cm above the plate, respectively. The sound pressures were measured over these two planes of the same dimension as 0.7 m × 0.7 m with a uniform spacing of 0.1 m for a total of 64 measuring points. Specifically, a linear microphone array consisting of eight microphones with an inter-element spacing of 0.1 m was employed. To acquire measurements across the entire region, the array was sequentially positioned at eight non-overlapping locations on the hologram plane situated 10 cm above the plate, effectively covering a total area of 0.7 m × 0.7 m and yielding 64 measurement points in total. During each acquisition, the transfer function between each microphone and the excitation loudspeaker input was estimated using the averaged periodogram method [34]. A consistent excitation signal was applied across all measurements, enabling the construction of a phase- and amplitude-consistent synchronized data set by multiplying the derived transfer functions with the known input signal. To validate the reconstruction accuracy, an additional set of measurements was conducted on a reconstruction plane located 5 cm above the vibrating plate, using the same measurement procedure as described above. This provided another set of 64 uniformly distributed sampling points, which were then used for comparison with the reconstruction results. The plane for the equivalent point sources was located 0.05 m behind the plate surface with the same plate size. The sound pressure data was collected by a calibrated (B&K type 4231) array of microphones (B&K type 4957), and they were logged and analyzed by a signal analyzer (B&K pulse). Before and during the experiment, the phases of all microphone arrays were also calibrated using the phase calibrator (B&K type 4231). Because the whole sound field could be considered perfectly coherent with the excitation signal, field pressures were referenced to only one fixed reference microphone. The following experimental steps were taken:

(1) The experimental bench and measuring device was installed.
(2) A signal was given to the vibration exciter separately and the pressure was measured on the hologram surface.
(3) The radiated pressure on the reconstruction plane was measured at 0.05 m above the steel plate as the actual value for comparison.
(4) After processing the corresponding data, it was imported into the analyzer, where the computer processed the analyzed results.

**Figure 10:**
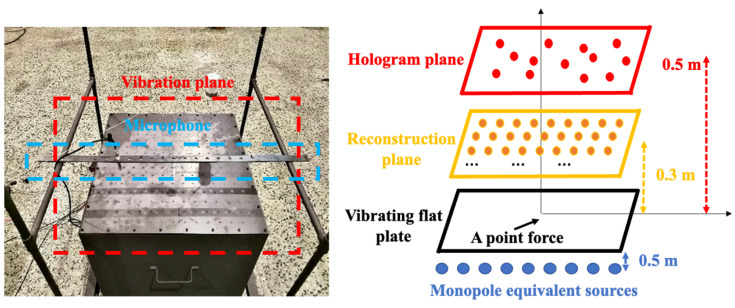
Setup description for vibrating plate experiment.

**Figure 11:**
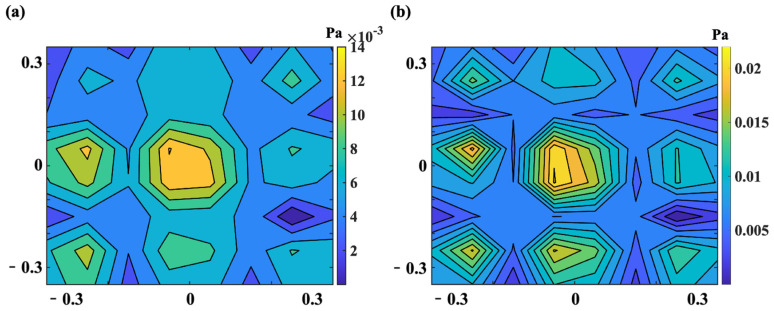
Distribution of measured actual pressure: (**a**) hologram plane, (**b**) reconstruction plane.

Figure 10 shows the schematic diagram of the vibrating plate experiment test, and Figure 11 shows the interpolated measurement pressure on the hologram and reconstruction planes at 250 Hz.

**Figure 12:**
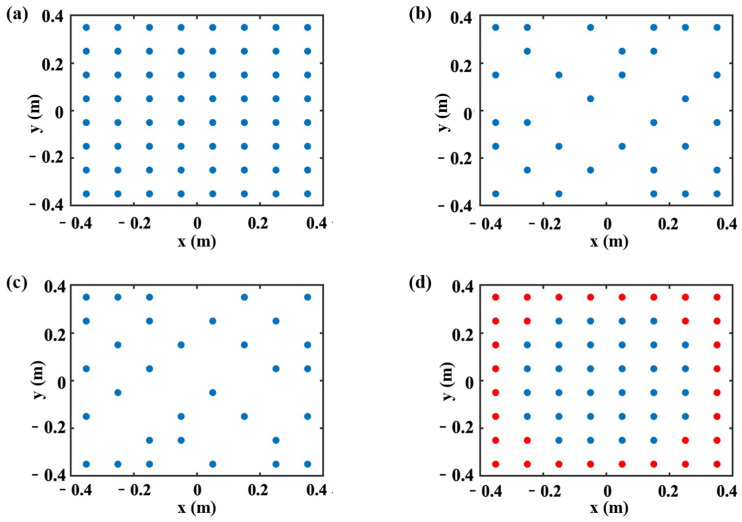
(**a**) Distribution of initial 64 microphones constituting the hologram plane, (**b**) 32 measurement points selected by the EfI method, (**c**) 32 measurement points selected by the MSV method, (**d**) 32 measurement points with 32 points of zero-padding. 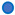 is the measurement points, and 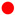 is the padding points.

Three data sets were prepared first to validate the findings in the preceding chapter. (1) Two sets of underdetermined hologram data were selected from the initial 64 evenly distributed candidate data by discarding the 32 data with small EfI values or large condition numbers. (2) In the third set, to test the zero-padding technique, 32 measured data in the middle part were selected from 64 evenly spaced points as initial data (0.5 m × 0.5 m with 0.1 m spacing), and the remaining 28 measuring points data were set as zero. Then, the patch method was implemented to reconstruct the 64 data points at evenly spaced positions. After several iterations, the iteration was stopped until reaching the iteration convergence condition, which was seven iterations in this test. Then, three sets of underdetermined hologram data were ready to be processed by the three regularization algorithms in a cross-linking way to reconstruct the pressure at 64 points on the reconstruction plane. Figure 12 depicts the 64 initial hologram data and the distribution of three underdetermined hologram data.

### 4.2. Test Result

**Figure 13:**
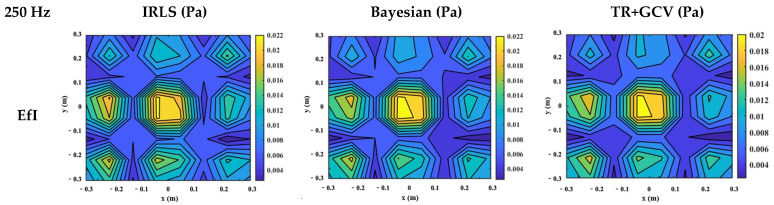

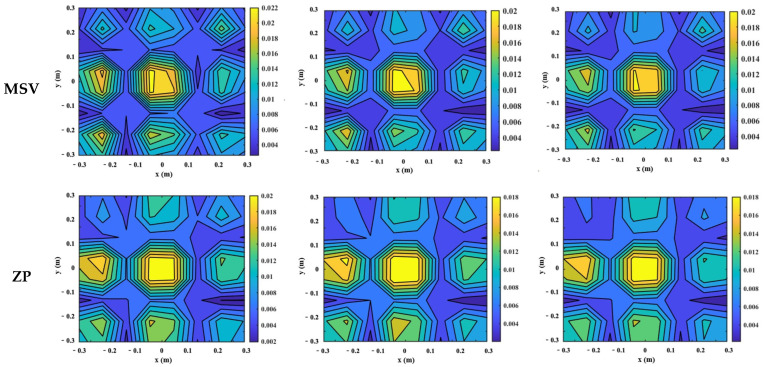
The reconstruction results from three underdetermined hologram data selected by three different choice schemes using three regularization algorithms (f = 250 Hz).

Figure 13 shows the reconstruction results from three underdetermined hologram data sets using the CS algorithm, Bayesian regularization, and TR + GCV regularization, respectively, at a sampling rate of 250 Hz. One can find that the reconstruction results of the patch with zero padding points generally reflect the actual situation of the sound field, but some details are directly ignored, no matter which regularization algorithm is used. This situation is most serious when using the TR + GCV regularization, making it impossible to provide an accurate result for the engineer. In contrast, the hologram data selected by the EfI technique or MSV method, combined with the CS algorithm, can provide a closer reconstruction to the actual one. The Bayesian regularization can also provide a better reconstruction result, although it is not as good as the CS algorithm. However, the reconstruction result combined with the TR-GCV regularization slightly differs from the actual one.

**Figure 14:**
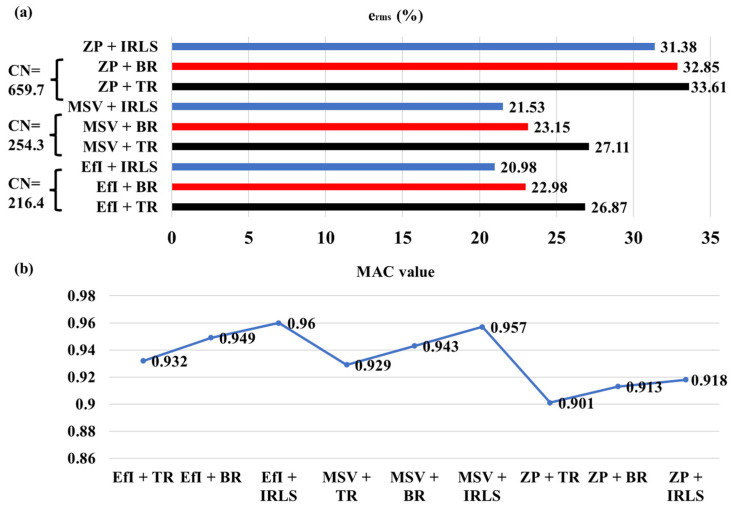
Summary of the reconstruction error in terms of (**a**) error norm and (**b**) MAC value (f = 250 Hz). The condition number of the transfer matrix is also indicated in (**a**).

Furthermore, Figure 14 summarizes the reconstruction result obtained from the cross-linked combinations of three selection methods of underdetermined hologram data and three regularization methods at 250 Hz. One can find that the reconstruction results of the patch with zero-padding points treated by the regularization are not bad from the viewpoint of MAC value. Still, the overall error level is larger than the other cases. When combined with the TR, it yields the worst output. The hologram data selected by the MSV method, combined with regularization, resulted in good reconstruction in general. In particular, when the MSV is combined with the CS algorithm, viz., IRLS, both reconstruction error and MAC value become very good. An excellent reconstruction result is obtained using the underdetermined data selected by the EfI technique, combined with any regularization method in general. Again, when the EfI technique is combined with the IRLS data compression method, it yields the best reconstruction results in both the overall reconstruction error and the reconstruction field distribution. It is noted that, for both EfI and MSV techniques, the combination with the BR can also improve the reconstruction results, although it is not as good as the CS algorithm. In Figure 11, it can be seen that the condition number of the transfer matrix established using the underdetermined hologram data and the EfI technique is less than half that of the other data selection methods. This means that the transfer matrix of the hologram data point selected by discarding the small EFI value bears a lower condition number, making it possible to encounter the convergence problem in the inverse process.

**Figure 15:**
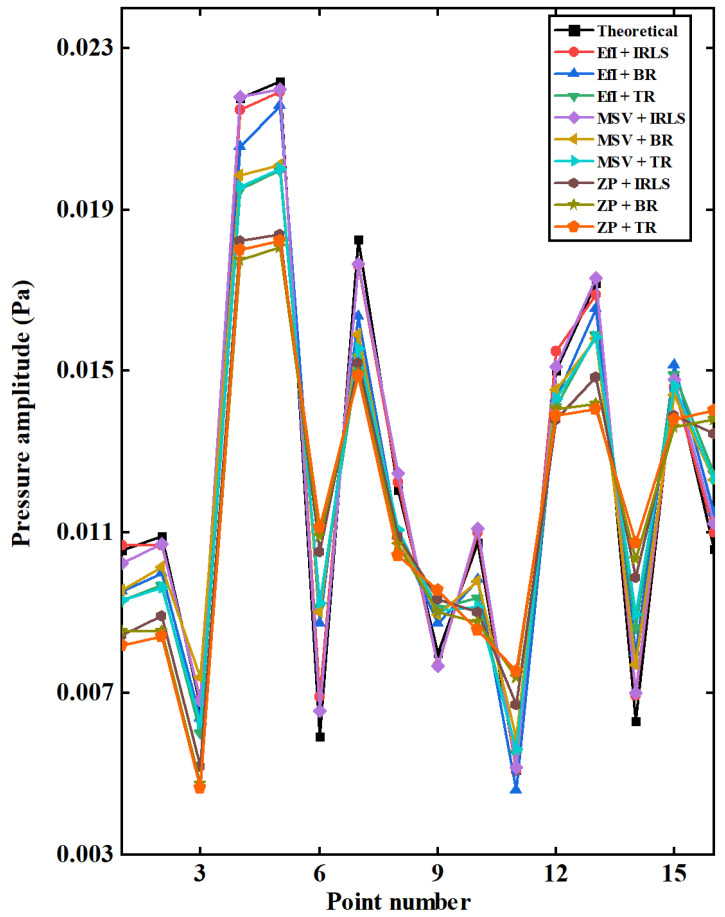
The comparison of the measured and reconstructed pressures at 16 points in the middle two columns of the reconstruction plane when 32 hologram data were used (f = 250 Hz).

The comparison between the reconstructed and theoretical pressures at 16 points in the middle two columns of the reconstruction plane at 250 Hz is shown in Figure 15. Again, one can confirm that the reconstruction results of underdetermined hologram data selected by the EfI or MSV method combined with the CS algorithm matches the measured values best. The reconstruction using the BR also only yields good results when it is combined with the EFI or MSV method for hologram data selection.

## 5. Conclusions

A method for enhancing the resolution of the reconstruction field based on underdetermined hologram data in NAH was studied by comparing cross-linked sets of hologram data selection methods and regularization techniques. The data selection methods used in the performance comparison were the EfI method, which eliminates the most dependent point sequentially; the MSV method, which removes the points sequentially, yielding minor singular values; and the data expansion method with zero padding (ZP), which is used with patch hologram data. The regularization methods used in the performance comparison were Tikhonov regularization (TR), statistical regularization based on the Bayesian technique (BR), and the data compression technique (CS). The comparison was made by cross-linking a data selection method and a regularization method.

Simulation studies with a vibrating plate revealed that the EfI and MSV methods yielded singularity factors with very similar magnitudes, unlike evenly spaced points or points with zero padding, which selected the equivalent of two or three times the number of hologram points selected by the EfI and MSV methods. Gradually decreasing the number of field points of an overdetermined hologram produced an underdetermined hologram. Then, consistent with the decreasing trend, the points in this hologram selected using the EfI or MSV methods had a much smaller condition number, minor reconstruction error, and larger modal assurance criterion (MAC) value than those obtained from the evenly spaced field points. Gradually reducing the number of field points in the EfI or MSV methods from the initially overdetermined data points did not improve the singularity factor significantly if the observed number of data points was overdetermined. However, underdetermined hologram data were obtained by further reducing the number of data points in this way, which led to dramatically improved reconstruction accuracy with the test frequency and model used in this study. In contrast, the evenly spaced points did not show a significant improvement when the same procedure was performed.

Cross-linking the sets of three hologram data selection methods and three regularization techniques, which gave nine cases, showed that the ZP technique and any regularization method generated the worst reconstruction accuracy and MAC value. In contrast, the EfI method combined with the data compression method yielded the best performance. The experimental results validated the simulation findings. They showed that the reconstruction error was drastically reduced (by at least 20–30% compared with that obtained using the ZP technique) when the underdetermined hologram data selected by the EfI and MSV methods were combined with any other regularization technique. When applied to either EfI or MSV techniques, the data compression method further reduced the reconstruction error by 20% relative to the TR method for the test example in this study. It was found that the best practice for preparing and inversely processing underdetermined hologram data is to use the EfI method for data selection and the data compression method for regularization.

This best practice for optimizing hologram measurement points provides a compact and reliable solution for many ill-posed acoustic problems. While our study focused on underdetermined sound field reconstruction, the proposed approach can be extended to a range of source identification tasks with limited measurements. It is particularly suitable for industrial and structural–acoustic diagnostics, where reduced sensor counts are essential. The method enables efficient reconstruction for applications such as mapping machinery noise, monitoring panel vibrations, and on-site sound field analysis. Furthermore, the approach holds strong potential in NVH applications, including cabin noise evaluation, panel contribution analysis, and tonal source localization in vehicles and mechanical systems, where accurate results are needed with minimal instrumentation.

## Figures and Tables

**Table 1:** First seven natural frequencies of the clamped 0.5 m × 0.5 m × 3 mm steel plate.

Mode Index	1	2	3	4	5	6	7
(m, n)	(1, 1)	(1, 2)	(2, 1)	(2, 2)	(2, 3)	(3, 2)	(3, 3)
Frequency (Hz)	59 Hz	147 Hz	147 Hz	235 Hz	385 Hz	385 Hz	528 Hz

## Data Availability

The raw data supporting the conclusions of this article will be madeavailable by the authors on request.

## Appendix A. Comparison of CPU Time

The calculations were performed on an HP EliteDesk 800 computer (manufactured by HP Inc., Palo Alto, CA, USA) equipped with an Intel Core i7-7700K processor (manufactured by Intel Corporation, Santa Clara, CA, USA). The CPU time for cross-linked sets of data selection and reconstruction methods is shown in Figure A1. The data includes the times required for preparing the measuring point (EFI technique, MSV method, and patch with zero-padding technique) and calculating the inverse problem using three regularization methods based on the prepared data. All three data selection methods require some time for data preparation due to the circular iteration process. In particular, the MSV method takes the longest time, regardless of the combined reconstruction algorithm. This is because it needs to calculate all the possible transfer matrices between the source and the field. The EFI technique summed up the time only for the SVD process. In contrast, the calculation time for the patch using the zero-padding technique is not short, as it requires complicated steps such as reconstruction, data replacement, and iterations until convergence. The IRSL algorithm takes a longer time than the TR and BR due to the longer iterations.
